# Acceptance Mindfulness-Trait as a Protective Factor for Post-Natal Depression: A Preliminary Research

**DOI:** 10.3390/ijerph19031545

**Published:** 2022-01-29

**Authors:** Dahlia Tharwat, Marion Trousselard, Dominique Fromage, Célia Belrose, Mélanie Balès, Anne-Laure Sutter-Dallay, Marie-Laure Ezto, Françoise Hurstel, Thierry Harvey, Solenne Martin, Cécile Vigier, Elisabeth Spitz, Anaïs M. Duffaud

**Affiliations:** 1 Groupe Hospitalier Diaconesses Croix Saint Simon, 75012 Paris, France; tharwatdahlia@hotmail.fr (D.T.); thdiaco@gmail.com (T.H.); 2 Unit of Stress Neurophysiology, French Armed Forces Biomedical Research Institute, BP73, 91223 Brétigny-sur-Orge, France; marion.trousselard@def.gouv.fr (M.T.); celia.belrose@gmail.com (C.B.); cecile.vigier@intradef.gouv.fr (C.V.); 3 APEMAC/EPSAM, Université de Lorraine, UR 4360, Ile du Saulcy, BP 30309, CEDEX 1, 57006 Metz, France; elisabeth.spitz@univ-lorraine.fr; 4 Réseau ABC des Psychotraumas, CEDEX 5, 34093 Montpellier, France; 5 French Military Health Service Academy, 1 Place Alphonse Laveran, CEDEX 05, 75230 Paris, France; dominique.fromage@gmail.com (D.F.); solenne.martin@intradef.gouv.fr (S.M.); 6 Périnatal Psychiatry Network, University Department of Child Psychaitry, CH Charles Perrens, 33076 Bordeaux, France; bales.melanie@yahoo.fr (M.B.); alsutter@ch-perrens.fr (A.-L.S.-D.); 7 BPHRC, Inserm 1219, Bordeaux University, 33000 Bordeaux, France; 8 CHR Mercy, Regional Hospital of Metz, 57000 Metz, France; ml.eszto@chr-metz-thionville.fr; 9 Protection Maternelle et Infantile du Department de la Moselle, 57000 Metz, France; francoise.hurstel@moselle.fr

**Keywords:** mindfulness-trait, postnatal depression, chronic stress, acceptance

## Abstract

(1) Background: the prevalence of postnatal depression (PND) reaches up to 20%. PND could be based on the interaction between a psychological vulnerability and chronic stress that pregnancy would activate. Vulnerability factors reflect a psychological profile mirroring mindfulness-trait (MT). A high level of MT is associated with an efficient regulation of both physiological and psychological stress, especially negative moods. Interestingly, mindfulness level can be improved by program based on mindfulness meditation. We hypothesize that MT is a protective factor for PND. We also postulate that negative moods increase during the pregnancy for women who develop a PND after delivery (2) Methods: we conducted a multicentric prospective longitudinal study including 85 women during their first trimester of their pregnancy and 72 from the childbirth to the baby’s first birthday”. At the inclusion, presence and acceptance of MT and various variables of personality and of psychological functioning were assessed. Mood evolution was monitored each month during the pregnancy and a delivery trauma risk was evaluated after delivery. PND detection was carried out at 48 h, 2, 6 and 12 months after the delivery with the Edinburgh Postnatal Depression Scale with a screening cut-off >11. (3) Results: high-acceptance MT is a protective factor for PND (OR: 0.79). Women without PND displayed less negative mood during pregnancy (*p* < 0.05 for Anxiety, Confusion and Anger). (4) Conclusions: these results suggest the value of deploying programs to enhance the level of mindfulness, especially in its acceptance dimension, before, during and after pregnancy, to reduce the risk of PND.

## 1. Introduction

Postnatal depression (PND) is the most common childbearing complication with a prevalence range of 5–25% in Western countries [1,2,3]. This prevalence differs between culture [2]. PND can have a broad range of depressive symptoms which varies in how severe they are. Symptoms may take some time to develop. It can start at any time within the first year after giving birth and may develop suddenly or gradually. This maternal pathology does not only affect the mother but the infant and family. The consequences for infants on the long-term emotional and cognitive development may be severe. Partner and family are also affected, thus PND appears to be a major public health problem [4]. The earlier it is recognized, diagnosed and treated, the faster the recovery will be. However, diagnosis of PND varies widely according to the methodological parameters. Currently, the Edinburgh Postnatal Depression Scale (EPDS) is the main screening tool widely used to detect, diagnose and assess the severity of the depression [5].

The mechanisms through which PND develop is not yet understood. During pregnancy and postpartum periods, the maternal organism undergoes remarkable biological, physical, social and emotional changes. Furthermore, an estimated 30% of pregnant women report psychosocial stress in their daily lives including job strain and depressive or anxiety symptoms [6]. Mounting evidence shows that the perinatal period is a time of increased vulnerability for mood disturbances and the onset of psychiatric disorders [7]. Altogether, the available data suggest that chronic stressors challenge women’ adaptability during the pregnancy and that the antenatal period offers a “window of opportunity” during which a preventative approach to this condition can be instituted. Although psychological and biological distress responses in pregnant women have been associated with poor fetal outcomes [8,9,10], little is known about the relationship between stress regulation ability and the risk of PND. Literature mainly focused on socio-psychological risk factors, among them personality factors, mediators of adaptation related to life event and to pregnancy, past-traumatic events or delivery trauma [1]. A particularly important risk factor would be a history of mood or anxiety disorder, especially having active symptoms during pregnancy [11]. Recently, the PND has been conceptualized from a biopsychosocial perspective [1]. 

In applied clinical psychology and psychiatry, the measurement of mood states is an important practical and behavior assessment consideration. Indeed, mood is less tightly linked to particular events than emotion and is thought to reflect the cumulative impact of multiple stressors (e.g., depression; [12]). The theory of mood postulates that mood involves a mechanism which monitors our physical and mental energy levels in relation to the perceived energy demands of our environment, and generates corresponding cognitive biases in our reasoning style, attention, memory, thought, and creativity [13,14]. Mood is likely to be relevant to understand behavior because it seems that subjects in a more positive mood state deal more easily with negative short-term experiences: a positive mood might therefore alleviate single negative events and stabilize emotional reactions. On the contrary, a negative mood state might taint experiences quite generally. For example, in sports, decreased Profile of Mood States vigor alert the coach to the risk of overtraining the athlete [15]. During intensified military training, depression ratings of participants suffering from the overtraining syndrome show a major increase, whereas depression ratings in healthy participants increase only little [16]. Keeping a careful watch for changes in mood can be useful in non-clinical population as such variations may reflect changes in others difficult to assess psychological states that are important. Mood states are thought to become particularly important and visible in situations of ambiguity and uncertainty where the expectation of a subject is pre-shaped to a smaller extent by the circumstances of the situation, as the pregnancy could be an example [17,18]. Furthermore, a body of literature suggests the interest of the dynamics of affective states to predict mood or sleep disorders, especially during pregnancy [19]. Since mood could represent the overall momentum, and its biasing is considered to influence on the perception of our environment, it is postulated that mood changes could reflect the potential dysfunctions of the adaption to our environment and that it might contribute to the symptoms of mood disorders [12,20,21]. Although few data on the follow-up of the mood of the pregnant woman are available, it seems relevant to better know the dynamics of the moods during the pregnancy as a follow-up that could reflect the cumulative impact of multiple stressors. 

Stress regulation during pregnancy has been mainly studied by focusing on negative abilities for validated risk factors of PND with neuroticism, assessed during pregnancy, clearly in relationship with a subsequent PND [8,9]. However, the positive psychology initiative, with its focus on the science of wellbeing and optimal human functioning, also suggests the importance of understanding protective factors to psychopathology. A pertinent protective factor that is understudied in the PND is the mindfulness trait (MT). MT characterizes the awareness that emerges through paying attention on purpose, in the present moment, and nonjudgmentally to the unfolding experience moment by moment [22]. Mindfulness has been conceptualized as a kind of trait, i.e., the ability to be mindful in one’s everyday life, regardless of events and stably in time [23,24]. MT refers to at least two dimensions: acceptance and presence [22,25]. The acceptance dimension consists of accepting inner events such as emotions, thoughts or beliefs when one feels them without judging it as either good or bad and without any reference to resignation [26]. Presence is the feeling of being present here and now. The presence feeling is based on an enhanced awareness of information that comes from within (body awareness and self-awareness), and outside the body (world awareness). As shown by mindfulness studies, positive associations were found between mindfulness and efficient emotional regulation, reduced perceived stress, and lower rates of negative mood and of psychological distress, including depression [27,28]. Its association with the Big Five personality traits [29] shows that MT mitigates negative reactivity tendencies associated with neurotiscism [30,31]. Furthermore, in line with the relevance of mood for dealing with uncertainty naturalistic environment, it has been showed that mindwandering and external distraction (all the more that the situation is stressful) are both manifestations of a common state of reduced attention focus that could participate to the level of negative mood. One of the processes of MT is that mindfulness functioning could help to deal with mindwandering and external distraction for maintaining a better level of positive mood and a lower level of negative mood [32,33]. Recently, it has been shown that MT enhances a positive childbirth experience which is known as reducing levels of postpartum depression [34]. Altogether, these studies suggest that MT could be a protective factor for PND. Interestingly, in line with the well-known benefits of mindfulness interventions for depression and relapses of depression [35,36,37], including in the general population [38], recent pilot studies showed that prenatal mindfulness interventions have potential benefits for improving the emergence of perinatal depression [39,40].

So far, no study has investigated the links between MT and the risk of PND. By taking into account the most usual psychological factors of PND and in line with the literature showing that MT is a protective factor to psychopathologies, we first hypothesized that a high level of MT would be a protective factor for PND. We further hypothesize that mindfulness may be the most important protective factor among the factors already studied. Furthermore, referring to the better emotion regulation associated to MT, our second hypothesis postulated that a high level of MT would be associated with less negative emotions during the pregnancy. The cumulative framework of chronic stress leads to the postulation that the increase in negative mood may come at the end of pregnancy. This exploratory hypothesis aims to evaluate the relevance of a follow up of the mood states during the pregnancy.

## 2. Materials and Methods

### 2.1. Participants

We conducted a multicentric prospective longitudinal study between May 2017 and March 2021 including 85 pregnant women. The study took place in three French hospitals: Pôle HFME, CHR Mercy, Metz—Maternité GH Diaconesses Croix St Simon, Paris—Centre Aliénor d’Aquitaine, CHU Pellegrin, Bordeaux. The women involved in the study had to meet the following requirements: (1) Inclusion criteria: before 17th week of gestation, pregnancy monitoring planned within one of the four facilities involved in the project, women over 18 years, covered by the French National Health Service and (2) Non-inclusion criteria: pathological pregnancy requiring increased medical monitoring, multiple pregnancy, ongoing pathologies at the time of inclusion: (i) immune or endocrine conditions; (ii) any psychological disorders (PTSD, depression or anxiety disorders, etc.); or (iii) neurological pathologies such as multiple sclerosis, hormonal or psychotropic drug therapies.

The study received prior approval from the Ile de France III Personal Protection Committee (21/06/2016; ID RCB: 2016-A00887-44; NTC 03088319). All women received information on the protocol and gave written consent prior to participation.

### 2.2. Protocole

This study followed pregnant women from the first four months of pregnancy up to 12 months’ post-birth. Each woman had to attend 10 visits: one visit within the first 16 weeks of pregnancy (inclusion visit (VI)), one visit every month between month 5 to month 9 of the pregnancy (VP5 to VP9), one visit within the 48 h of the birth (VB1) and three visits 2, 6 and 12 months after delivery (VB2, VB3 and VB4, respectively). Except the last three VB visits which has been performed remotely, each visit was realized at the hospital by a midwife or a clinical research assistant. During each visit, participant had to complete different questionnaires, see Table 1 and Table 2 for detailed description. The last three VB visits were remotely performed.

### 2.3. Variables

#### 2.3.1. Sociodemographic Data

Sociodemographic information included age, social environment, professional and marital status, and the number of children. We also asked the women about their history of psychological care or support and their personal and familial history of depression. Questions about their pregnancy history and the condition of the current pregnancy were also asked.

#### 2.3.2. Three Main Questionnaires

- For the PND status: PND was assessed using the Edinburgh Postnatal Depression Scale (EPDS) with a screening cut-off >11 [41]. French validation showed good psychometric qualities [42]. The EPDS is a 10-item self-report questionnaire assessing the symptoms of depression and anxiety. Each self-descriptive statement about the 7 last days was evaluated using a four-point Likert scale ranging from 0 (no change from usual) to 3 (an important change from usual). It was fulfilled four times at VB1, VB2, VB3 and VB4.
- For the mindfulness evaluation: the 14-item, self-administered Freiburg Mindfulness Inventory short form (FMI short form) assessed mindfulness [25] developed for people without any background knowledge in mindfulness [25]. French validation showed good psychometric qualities [43]. It constitutes a consistent and reliable scale evaluating several important aspects of mindfulness, which indexes trait mindfulness as presence and nonjudgmental acceptance [25]. Each self-descriptive statement was evaluated using a four-point Likert scale ranging from 1 (strongly disagree) to 4 (strongly agree). Depending on the suggested time frame, state-and trait-like components could be assessed. In the present study, the short form was used for measuring MT. It was fulfilled once at VIN.
- For the mood follow-up: The mood was evaluated at the beginning of both sessions using an abbreviated version of the Profile of Mood States (POMS) [44]. French version replicated the English initial validation [45]. Abbreviated version of the POMS consisted in an adjective checklist of 37 items rated on a five-point scale that ranges from 1 (not at all) to 5 (extremely). The subjects were asked to answer according to their present mood. Six factors were then calculated: anxiety-tension, depression-dejection, anger-hostility, fatigue-inertia, vigor-activity and confusion-bewilderment. It was fulfilled during the pregnancy from VI to VP9, unless the woman gave birth before 9 months of pregnancy. Given that pregnancy produce a multitude of affective changes, the use of multidimensional mood model (not only positive or negative), as the POMS does, appears relevant.

#### 2.3.3. Four Questionnaires for the General Psychological Functioning

- Cloninger’s Temperament and Character Inventory-Revised (TCI-R) short-version is a 56 items self-report questionnaire with 5-grade Likert scale responses ranging from definitely false to true [46,47,48]. It is intended to assess the individual differences of the four temperaments (Harm Avoidance, Novelty Seeking, Reward Dependence and Persistence) and three character higher-order dimensions (Self-Directedness, Cooperativeness and Self- Transcendence). Each higher order dimension is further divided into sub-scales. It is considered as a useful instrument to assess Cloninger’s model of the 7 dimensions of personality in non-clinical samples [46,47,48].
- Anxiety-trait level was assessed using the French version of the Spielberger State-Trait-Anxiety Inventory (S-STAI; [49,50]). The 20 items of the trait subscale ask subjects to indicate the intensity of their anxiety in general. In this study and the sample was categorized in three groups according to their score [36]: women with a high score (score > 65), women with a middle score of trait-anxious-trait (56 < score ≤ 65), and women with a low score (score < 56).
- The Warwick-Edinburgh Mental Well-being Scale (WEMWBS, [51,52]) covers both hedonic constructs including the subjective experience of happiness and life satisfaction, and eudaemonic constructs addressing psychological functioning and self-realization in the previous two weeks [51]. It comprises 14 items and responses are made on a 5-point scale ranging from “none of the time” to “all of the time”. The scale is suitable for monitoring mental well-being in healthy populations as it shows few ceiling or floor effects [51].
- The Symptom Checklist-90 revised (SCL90R, [53]), is a common mental health evaluation tool used to assess psychological problems. Each item is scored on a scale from 0 (“not at all”) to 4 (extremely”) based on how much an individual was bothered by each item in the last weeks. Five symptoms’ dimensions were evaluated: somatization—, obsessive-compulsive—OC, interpersonal sensitivity—IS, depression—D, and anxiety—A.
- Four homemade analogic visual scales (from 0 “very bad/low” to 10 “very good/high”) were used for quality of life assessment: (1) ”in the past month, how would you rate the quality of your sleep?”, (2) “in the past month, how would you rate your stress level at work?”, (3) “ in the past month, how would you rate your level of stress at home?” and (4) “in the past month, how would you rate your level of apprehension about giving birth?”

#### 2.3.4. Four Questionnaires for the Specific Pregnancy and Delivery Psychological Functioning

- The Prenatal Attachment Inventory (PAI, [54]) is a 21 items questionnaire for expectant mothers which assess maternal-fetal attachment defined as the strength of mothers’ emotional ties with the fetus (and also known as prenatal bonding. It captures variability in expectant mothers’ behaviours, cognitions and emotions towards the fetus, which appear important for positive prenatal health practices [55]. Expectant women were asked to assess how often they engaged in specific thoughts or behaviours towards the fetus on a 4-point scale (1 « almost never » to 4 « almost always »).
- The Questionnaire de Dépistage Anténatal du risque de Dépression du Postpartum (DAD-P; postpartum Depression Risk Screening Questionnaire), previously named le Questionnaire de Genève, was used to detect women at risk to develop PND already during pregnancy [56]. It is based on 10 items, six for screening, and four supplementary items for optimizing the screening leading to several screening strategies, depending on whether broad or targeted screening is.
- The Multidimensional Scale of Perceived Social Support (MSPSS) is a 12 items questionnaire assessing the perceived social support from three sources: Family, Friends, and a Significant Other [57]. A seven-point Likert-type scale ranging from “strongly disagree (1)” to “strongly agree (7)” was used with a total score obtained by adding the score for each statement, divided by the total number of statements. It was validated for expectant women [58].
- The Labour Agentry Scale (LAS) is a 29 items instrument measuring expectancies and experiences of personal control during childbirth [59]. It consists of short affirmative statements (e.g., ‘I felt confident’ and ‘I felt tense’). Women were asked to rate each statement on a seven-point Likert scale from 1 (representing rarely) to 7 (representing almost always).

#### 2.3.5. Three Questionnaires for Delivery Trauma and Post-Traumatic Stress Disorder (PTSD)

- The traumatic event scale (TES) was a 21 items questionnaire developed in accordance with DSM-IV criteria for the PTSD syndrome and comprises all the DSM-IVR symptoms and criteria of PTSD [60,61]. The TES was divided on two parts: the part one (TES 1) quantifies frequency and severity of single posttraumatic stress symptoms focusing on childbird; part two (TES 2) assesses how each of the 21 symptoms impacts the daily quality of life.
- The Peritraumatic Dissociative Experiences Questionnaire (PDEQ) is 10-items self-questionnaire assessing peritraumatic dissociation that occurred at the time of a trauma [62,63]. Dissociation is well-recognized as a risk factor for developing PTSD. A five-point Likert-type scale ranging from “not true (1)” to “totally true (5)” was used. A score greater than or equal to 22 attests to the presence of clinically significant peri-traumatic dissociation [64].
- The traumatic impact of childbirth questionnaire (ITA) assesses the recollection of experience during labour and delivery. It is constituted first, by 18 items rated from 1 to 7 (ITA-1), then, by 14 items rated from 1 to 6(ITA-2). This specific questionnaire has not yet been validated.

### 2.4. Statistical Analysis

**Figure 1:**
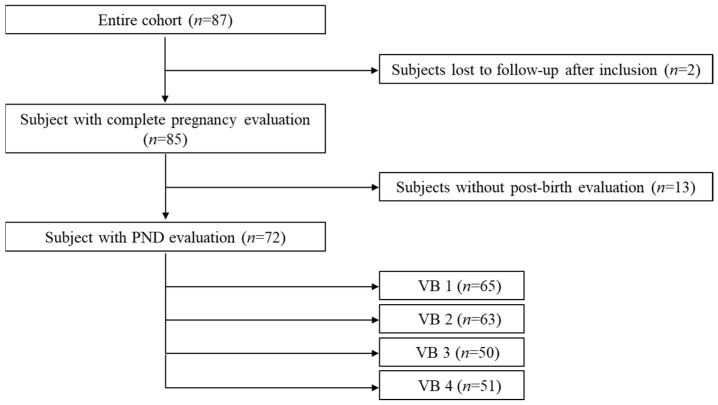
Flow diagram showing subpopulations within the initial cohort with the sample size at each of the four VB sessions.

All statistical analyses were performed using SPSS software (SPSS INC, Chicago, IL, USA, version 24.0) and Stata software (Stata corp, College Station, Texas, USA, v.14). The reliability of each psychological measurement (self-administered questionnaire) was gauged by computing Cronbach’s alpha and all alphas were above 0.74, which indicates good reliability. The description of the population was complemented by a flow chart showing PND trajectories (Figure 1).

For the description of the sample at VI, we have completed the presentation of the sociobiographical data by separate comparisons on MT score at VI and POMS sub-scores at VI according to the PND status after delivery. Each comparison was applied using t-test or Mann-Whitney test if normal distribution was not found. PND status was evaluated using a score above 11 on the EPDS that is recognized as the sensitive and specific threshold for PND [28]. The PDN + group consisted of women with a score on the EPDS above 11 at least at one of the post-birth visits (VB1, VB2, VB3 or VB4). The PND - group consisted of women with EPDS score always below the threshold (11) whatever the post-birth visits.

For the first aim of the study, multivariate logistic regressions were used for evaluated the risk factors of the PND from variables that were recorded either at VI (SD, FMI, TCI-R, S-STAI, WEMWBS, SCL90, DAD-P), or during pregnancy (VG mean scores for each of the sub-factors of the POMS, of the MSPSS and at VB1 (LAS, TES, PDEQ and ITA) (Table 1 and Table 2). The variables significant at the 20% threshold in univariate (Table 5) were added to the multivariate model and then a backward selection was carried out until the final model with variables significant at the 5% threshold was obtained (Table 6). We calculated unadjusted odds ratios for significant variables. The fit to the statistical model verified by analysis of the residuals and the Hosmer-Lemeshow goodness-of-fit test.

For the second aim of the study, separate inter-group (PND + versus PND −) comparisons were carried out on each mood variable using repeated measures analysis of variances (ANOVAs) or Mann-Whitney test at each session of the pregnancy if normal distribution and homogeneity of variance were not found.

The significance level was set at *p* ≤ 0.05.

## 3. Results

### 3.1. Population

The studied cohort (*n* = 72) was extracted from the initial population (*n* = 85) by taking into account the women who had at least one EPDS assessment post-birth (Figure 1). The number of women with PND + was assessed among the 72 women with a response on the EPDS at least at one of the post-birth visits (VB1, VB2, VB3 or VB4). At VB1, 65 women completed the EDPS and 12 had a score above 11 (18.46%); at VB2, 63 women completed the EDPS and 12 had a score above 11 (19.05%); at VB3, 50 women completed the EDPS and 9 had a score above 11 (18%); and at VB4, 51 women completed the EDPS and 7 had a score above 11 (13.72%).

Taking into account the women who had at least one score strictly higher than 11 on the EPDS during the postpartum follow-up, 26 women had an episode of PND (PND+). There was no difference between the 3 centers in terms of the number of PNDs per center in relation to the number of women included in each center.

Socio-demographic characteristics of the initial cohort are summarized in Table 3.

### 3.2. PND

In accordance with the flow chart, comparisons were applied at VI between the two groups (PND + and PND −) for the FMI and POMS key questionnaires for the cohort of 72 women who had at least one EPDS assessment post-birth. As showed in Table 4, significant differences were found for mindfulness acceptance and mindfulness total mean scores. Except for confusion-bewilderment mood subscale, no difference was observed in terms of mood at inclusion.

### 3.3. Risk Factors for the PND

Among the 72 women who had at least one EPDS accessment post-birth, the univariate analysis showed that among the categorical explanatory variables (i.e., sociodemographic variables), none were significant except for the history of psychological care or support. It also showed the quantitative variables of interest (Table 5) among them FMI-acceptance and FMI total scores.

The multivariate analysis highlighted odds ratios for unadjusted variables with the significant results Table 6.

Finally, the index plot does not detect any outliers or suspicious structuring and the linear prediction/residuals graph is satisfactory. The Hosmer-Lemeshow goodness-of-fit test is compatible with the data (*p* > 0.05).

### 3.4. Mood Evolution during the Pregnancy According to PND Status

**Figure 2:**
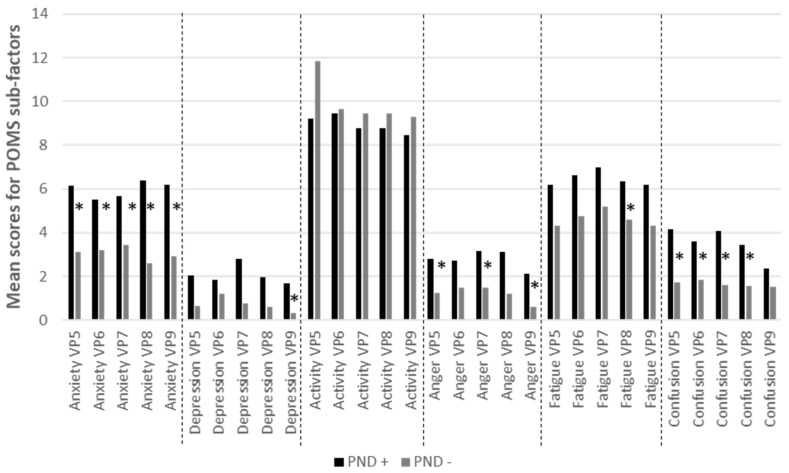
Differences between the two PND groups in their POMS mean sub-scores. Differences were tested with a Mann-Whitney test, *: *p* < 0.05.

Overall, Mann-Whitney tests applied at each session among the 72 women who had at least one EPDS assessment post-birth showed that the PND + group had more negative moods than the PND- group. The significant differences observed are displayed in Figure 2. at each session of the pregnancy if normal distribution and homogeneity of variance were not found Briefly, DPN+ women appeared to have significantly higher score in Anxiety (at each time point), Confusion (4 time points out of 5) and Anger (3 time points out of 5).

Many trends towards a difference between the two groups are observed: VP7 depression (*p* = 0.054), VP8 depression (*p* = 0.076), VP6 fatigue (*p* = 0.063), VI confusion (*p* = 0.062), and VP9 confusion (*p* = 0.087). No difference was observed for activity-vigor measures during pregnancy.

## 4. Discussion

This study investigated the links between MD and the risk of PND in physiological pregnancy with the main hypothesis that MD would constitute a protective factor for the risk of PND. Results are partially in accordance with this hypothesis by showing that the acceptance dimension of MD is the only pregnancy-related protective factor.

Acceptance is characterized by an absence of resignation when perceiving one’s own experience through an attitude of acknowledging the experience rather than judging it as either good or bad. Although empirical research has mainly operationalized mindfulness as a unidimensional construct, or as a presence ability, research on the acceptance dimension has so far been limited. The exact nature of acceptance is not yes understood. Shedding further light on the differential roles of presence and acceptance would contribute to disentangling the processes through which mindfulness impacts pregnancy mental effects on women [65]. Thus, the acceptance dimension of the MD could actually act as an emotional buffer that allows reaction without overinterpreting the stressful situation induced by the pregnancy, the anticipation of delivery or the delivery itself. Furthermore, it can be proposed that acceptance prevent women who have suffered from losses or threats because of pregnancy as suggest by the absence of effect of having an unplanned pregnancy. This hypothesis needs further study for validation. Acceptance could also prevent women who have been psychologically weakened by them from reacting less judiciously to situations that arise in the future as suggested by the absence of effect of being pregnant for the first time or not. From this perspective, the mindfulness acceptance disposition could serve as a protective factor by providing access to beneficial resources during the process of pregnancy. This is suggested by the absence of protective effect of the perceived social support that the results highlighted. In order to face many changes and adaptations, mainly outside the woman’s control (e.g., bodily changes and physical complaints) that occur during pregnancy, different psychological processes can be called upon. It is not intended to be exhaustive, but rather to suggest processes that the limited existing literature on acceptance has already shown. Acceptance could first allow access to the feeling of self-efficacy [66] for dealing with the changes that a pregnancy implies. Given the relationship between flexibility and mindfulness for emotion regulation [67], acceptance could further foster flexibility and this would help women to cope with the singular time of the pregnancy and the stressful situation of delivery. Furthermore, fully conscious acceptance, i.e., being aware of one’s painful thoughts and emotions as they are, without trying to suppress or avoid them has been recently studied by the concept of self-compassion [68]. Several studies have reported the usefulness of self-compassion in the face of difficult situations: for example, it allows a better stress management by acting on biopsychosocial responses [69], protects against depression and negative affect [70,71,72].

Results also identified risk factors for PND. They highlighted two main risk factors by showing that the obsessive-compulsive dimension of the Symptom Checklist-90 and having an history of psychological care increases by 3 to 4 the risk of triggering a PND. Obsessive-compulsive disorder (OCD) has its own chapter (obsessive–compulsive and related disorders) in DSM-5 and is no longer considered an anxiety disorder (APA; 61). It is characterized by a combination of intrusive thoughts and ritualistic behaviors carried out in an attempt to allay the anxiety associated with these thoughts. The general community life-time prevalence of OCD throughout the world is approximately 1% whereas a higher rate of 2–3% is observed in pregnant and postpartum women [61]. Higher rates among women in the postpartum period were sometimes described with a prevalence of 11% at 2 weeks postpartum [73,74]. Almost half of these women had persistence of symptoms at 6 months postpartum [73,74]. Clinical studies of OCD indicate that symptoms are stress-responsive [73,74,75]. This suggests that stressful situations, such as pregnancy and the postpartum period, may exacerbate or perhaps even predispose women to OCD symptoms. The postpartum period appears to be a high-risk time for the development of OCD symptoms [76]. Although the overlap between OCD and depression is still a matter of debate, both in terms of the mechanisms involved and the risk factors [77], obsessive-compulsive personality disorder symptoms could be a risk factor for postpartum depressive symptoms [66,67,68,69,70,71,72,73,74,75,76,77,78]. If additional data reinforce this hypothesis, it suggests that an assessment of OCD or obsessive-compulsive personality disorder may be useful for screening pregnant women for PND risk. Our results found that a simple obsessive-compulsive symptoms evaluation, collected no later than the 4th month of pregnancy, lead to a relevant screening for PND risk. This result was in line with the influence of having an history of psychological care, which underlies a vulnerability profile. Indeed, findings have suggested some potential psychological vulnerability factors for development of obsessive-compulsive symptoms [79,80]. With an effect of the culture, the vulnerability profile includes cognitive factors of appraisal and thought control, religiosity, self-esteem and personality characteristics such as neuroticism [80]. This psychological vulnerability could be related to a physiological stress vulnerability [80,81]. Mothers showing a low-level parasympathetic activity may have fewer emotional, physiological, and psychological resources for dealing with the challenge of the post-birth [81,82,83]. Interestingly, this profile of vulnerability did not include risk factors related to the experience of pregnancy such as the prenatal attachment, prenatal depression risk or the labour agentry. Likewise, trauma as experience of traumatic delivery was not found to be risk factor of PND.

In accordance with our second hypothesis and given the relationship between acceptance and emotional regulation [71], we observed differences in negative mood level during pregnancy between women with PND and healthy women. All negative mood, including anxiety-tension, depression-dejection, anger-hostility, fatigue-inertia, and confusion-bewilderment were higher for PND+ whereas no difference was found in vigor-activity mood. The differences mainly appeared after the first trimester of the pregnancy with a peak in the last months of the pregnancy, except for the anger-hostility mood which was higher for the PND+ group compared to the PND- group at the inclusion visit. Anger is an emotional state or mood according to the duration of stat which is considered as the tip of the iceberg, while other underlying negative emotions, such as fear, disappointment, and anxiety, sometimes induce anger [84]. Anger is associated with cognitive distortions, verbal and motor behavior, and patterns of physical arousal and then depression [84]. More precisely, personal trait anger (i.e., high trait anger) lead to internalized anger and not expressing themselves, processes which increase the risk of depression [85,86]. In line, previous studies have found a clear relationship between maternal anger in post-natal and PND [85,86,87,88]. Pregnant women who have high trait anger tend to have greater difficulty in regulating their emotions, including anger, for dealing with the post-natal lifestyle changes [88,89]. However, anger during pregnancy has less been studied. In the last trimester, resilience was found as one of the mediator between trait anger and PND [89]. Our results suggest that anger expression is present from the first trimester of pregnancy in the PDN-to-be women. Interestingly, on one hand, previous work has suggested that angry and aggressive tendencies may be reduced in individuals with higher levels of dispositional mindfulness because of their better ability to regulate emotions [90,91], including acceptance awareness ability [92]. On the other hand, higher trait anger and anger suppression was reported for patients with OCD and was associated with non-acceptance of negative emotions compared to non-OCD subjects [90,91,92]. Mindfulness acceptance would be a protective factor for PND by allowing a better regulation and expression of anger. In addition, on a clinical point of view, mood assessment could be useful to screen for low levels of suffering that may or may not be an indicator of PND risk. Whether or not mood is a good marker of a risk of progressing to PND need further research. Nevertheless, mood assessment is neither stigmatizing nor anxiety-provoking. In addition to taking into account small changes in mood during pregnancy may simply allow the initiation of self-confidence to rely on perinatal professionals in order to better live the pregnancy.

Main limitations of this study concern the sample and the method. First, our sample is small and results need to be confirmed by further studies. Moreover, those preliminary results are only applicable to women with physiological pregnancy, since women with pathological pregnancy (as defined by requiring increased medical monitoring, multiple pregnancy, ongoing pathologies at the time of inclusion) were excluded. Second, we only used to the Cloninger’s Temperament and Character Inventory-Revise which assesses the individual differences of the four temperaments (Harm Avoidance, Novelty Seeking, Reward Dependence and Persistence) and three character higher-order dimensions (Self-Directedness, Cooperativeness and Self- Transcendence). We cannot infer trait anger or obsessive-compulsive type personality disorders. Third, the history of psychological treatment which is found to be a risk factor to PND was not studied in detail. It would be relevant to ask women more information about the psychological help that they received as how long did it last for better understanding this risk factor in its relationship with PND. Fourth, the statistical analyses were based on many questionnaires which is a weakness of the study but since it was an explanatory study, statistics were conducted without any preconception. Furthermore, we collected rather few sociodemographic variables compared to the number of psychometric variables. Finally, PND status was evaluated using a score above 11 on the EPDS at least at one of the post-birth visits (VB1, VB2, VB3 or VB4). This choice of categorization based on a single score above the threshold may have overestimated the number of women with PND. PND + status could have been confirmed by a clinician or decided after two successive measurements above the threshold.

## 5. Conclusions

This study highlights that women with a high level of acceptance were less at risk of developing a PND whereas women with a vulnerability profile including obsessive-compulsive symptoms and an history of psychological care before the pregnancy were more at risk of PND. Furthermore, anger mood was present at the beginning of the pregnancy for women who develop a PND. Further studies should be undertaken to focus on assessing the acceptance and presence dimensions more specifically in order to continue disentangling their potential different effects on PND risk.

Interestingly, mindfulness practices improve one’s ability to be mindful [93]. Mindfulness practices rely on training one’s mind to sustain attention on bodily experiences, primarily breathing, and deliberately return one’s attention to these experiences whenever they are distracted [94]. Recent studies found a benefit effect on mindfulness practices as a valuable preparation for the challenges they met during pregnancy, childbirth and parenthood [95] and as a prevention for PND in the perinatal period [96].

However, there is currently a real need to evaluate the benefit of mindfulness training during pregnancy. Practices that develop an individual’s capacity to be mindful have been individualized in the form of intervention programs. Meditation consists of concentrating on one’s sensations during breathing exercises, which then represent an attentional baseline, thus making it possible to notify any other body sensation in relation to this baseline. Among these practices, we should note "mindfulness-based stress reduction" (MBSR) [39,93] and the "mindfulness-based cognitive therapy" (MBCT) [97]. These first programs were used to focus on the management of chronic pain, stress and then depression. Training programs, as ACT (Acceptance Commitment Therapy; [67,98,99]), could be used preferentially to develop the acceptance dimension during pregnancy by focusing on developing the ability to focus on one’s own experience for dealing with the stress of the pregnancy. They also could be used as a psychological support for the stress of delivery as for the stress and daily worries that appear after delivery.

## Figures and Tables

**Table 1:** Synopsis of study design. Study timeline for each participant.

	**1st** **Trimester**			**2nd** **Trimester**			**3rd** **Trimester**			**Delivery**												
Month	1	2	3	4	5	6	7	8	9		1	2	3	4	5	6	7	8	9	10	11	12
Visit	VI				VP5	VP6	VP7	VP8	VP9		VB1	VB2				VB3						VB4

**Table 2:** Synopsis of study design. Details of visit requirements. Each participant will have to attend 10 visits from their 1st trimester of pregnancy up to 12 months post-delivery. VI: inclusion visit, VP: pregnancy visits from the 4th month to the 9th month of pregnancy, VB: post-birth visits 48 h, 2, 8 and 12 months post-delivery. FMI: Freiburg Mindfulness Inventory, POMS: Profile of Mood Scale, EPDS: Edinburgh Postnatal Depression Scale, TCI-R: Temperament and Character Inventory-Revised, STAI: State-Trait Anxiety Inventory (Trait version), WEMWBS: Warwick-Edinburgh Mental Well-being Scale, SCL-90: Symptom Checklist-90 revised, QoL: Quality of Life, PAI: Prenatal Attachment Inventory, DAD-P: postpartum depression risk screening questionnaire, MSPSS: Multidimensional Scale of Perceived Social Support, LAS: Labour Agentry Scale, TES: Trauma Event Scale, PDEQ: Peritraumatic Dissociative Experiences Questionnaire, ITA: traumatic impact of childbirth questionnaire. The “x” mark indicates when the questionnaire was completed.

		Pregnancy						Post-Birth			
		Inclusion Visit VI First Trimester	Monthly Visit During Trimester 2 and 3					Follow-Up Visit Post Delivery			
			VP5	VP6	VP7	VP8	VP9	VB1	VB2	VB3	VB4
Regulatory information	Notice—Consent form	x									
Keys-questionnaires	Socio-demographic information	x									
	FMI	x									
	POMS	x	x	x	x	x	x				
	EPDS							x	x	x	x
General psychological functioning	TCI-R	x									
	STAI	x									
	WEMWBM	x									
	SCL-90	x									
	QoL	x	x	x	x	x	x				
Specific psychological pregnancy and delivery functioning	PAI	x	x	x	x	x	x				
	DAD-P	x									
	MSPSS	x	x	x	x	x	x				
	LAS							x			
Trauma and post-traumatic stress disorder questionnaires	TES							x			
	PDEQ							x			
	ITA							x			
Duration of questionnaires (minutes)		120	15	15	15	15	15	30	5	5	5

**Table 3:** Summary of the socio demographic data of the initial cohort.

	Characteristics		
Age	32.4 +/− 4.5 years (29.0–35.0)		
Pregnancy Age at the Inclusion	16.7 Weeks of Amenorrhea (WA) +/−2.0 WA (15.2–18.0 WA)		
		***n***	***%***
Site repartition	Paris	43	50.6
	Bordeaux	32	37.6
	Metz	10	11.8
Marital status	Couple	81	96.4
	Couple but single living	3	3.6
	Geographical celibacy	1	0.01
	Missing data	1	0.01
Historic of psychological care	Psychological support	51	60
	No psychological support	34	40
Number of children	No child	57	67.9
	One child	22	26.2
	Two or more children	6	6
Previous pregnancies	None	48	56.5
	Previous pregnancy *	15	17.6
	Two or more pregnancies	22	25.8
Type of pregnancy	Spontaneous	79	92.9
	Medically assisted reproduction:	6	7.1
Desired pregnancy	Yes	74	88.1
	No	10	11.9
	Missing data	1	0.01

*: (including spontaneous miscarriage, voluntary termination of pregnancy, and medical termination of pregnancy).

**Table 4:** Differences between PND + and PND − for the FMI and POMS key questionnaires at VI in the sample of 72 women.

	Variables	DPN + Mean (SD)	DPN − Mean (SD)	*p*-Value *
Freiburg Mindfulness Questionnaire (FMI)	Presence	17.5(3)	18(2.6)	0.52
	Acceptance	19.6(3.1)	212(3.5)	0.003
	Total	37.1(5)	40(5.3)	0.03
Profile of Mood Scale (POMS)	Anxiety-tension	6(5.5)	3.8(3.8)	0.08
	Anger-hostility	3.2(3.7)	1.8(3.7)	0.11
	Depression	1.7(2.22)	1.3(2.5)	0.5
	Fatigue-inertia	5.5(4.7)	5(3.8)	0.63
	Activity-vigor	11.3(4.1)	11.8(3.8)	0.6
	Confusion-bewilderment	3(3)	1.5(1.9)	0.03

*: *t*-test.

**Table 5:** The associations between potential predictors, measured at V1, VG or VP1, and postnatal depression, determined in univariate regression analyses. Only predictors that were significant at the 20% threshold in previous analyses are included.

Sessions	Variables *	Odds-Ratio	IC 95 %	*p*-Value
VI	FMI_acceptance	0.9	0.66–0.93	0.003
	FMI_total	0.90	0.80–0.99	0.025
	TCI_ Self-Directedness	1.14	1.03–1.26	0.010
	TCI_ Cooperativeness	1.13	0.97–1.35	0.120
	POMS_tension_anxiety	1.11	1.00–1.25	0.050
	POMS_ anger-hostility	1.11	0.98–1.28	0.110
	POMS_ confusion-bewilderment	1.29	1.05–1.63	0.013
	STAI-Trait			0.034
	Very low	—	—	
	Low	4.52	1.25–21.76	0.033
	Middle	8.87	1.80–54.92	0.011
	High	6.33	0.21–194.45	0.232
	WEMWBS	0.94	0.87–1.02	0.127
	SCL_obsessive-compulsive,	4.21	1.75–11.93	0.001
	SCL_ interpersonal sensitivity	2.57	1.03–7.34	0.042
	SCL_depression	3.21	1.39–8.67	0.005
	SCL_anxiety	2.32	1.00–6.20	0.050
	SCL_hostility	3.28	1.13–11.11	0.028
	SCL_Phobic anxiety	4.12	0.57–40.20	0.160
	SCL_Paranoid ideation	2.71	0.76–10.86	0.124
	SCL_Psychoticism	5.30	1.49–32.65	0.005
	SCL_General Severity Index	6.38	1.76–31.34	0.003
	SCL_Positive Symptom Total	1.04	1.01–1.08	0.012
	SCL_, Positive Symptom Distress Index	0.72	0.52–0.93	0.012
	MSPSS_Friends	0.70	0.41–1.16	0.165
	MSPSS_total	0.61	0.32–1.11	0.104
	QoL_ level of stress at work	1.16	1.01–1.38	0.038
	Age	0.93	0.82–1.03	0.178
	History of psychological care			0.006
	No	—	—	
	Yes	4.06	1.50–11.62	0.006
	Number of children			0.018
	0	—	—	
	1	0.30	0.06–1.07	0.086
	More than 2	6.44	0.87–131.50	0.108
VG **	POMS_tension_anxiety	1.41	1.15–1.79	<0.001
	Prenatal Attachment Inventory	0.94	0.88–1.00	0.066
	MSPSS_Friends	0.67	0.38–1.12	0.128
	MSPSS_total	0.57	0.29–1.09	0.088
	QoL_ quality of sleep	0.77	0.58–0.98	0.037
	QoL_level of stress at work	1.25	1.02–1.56	0.029
	QoL_level of stress at home	1.49	1.15–2.01	0.002
	PND Risk Screening Questionnaire			0.175
	Having no risk	—	—	
	Having a risk	3.47	0.57–23.78	0.180
VP1	Traumatic event scale 1	1.16	0.99–1.39	0.068
	Traumatic event scale 2	1.31	1.12–1.62	<0.001
	ITA_1	1.07	1.03–1.12	<0.001
	ITA_2	1.09	1.03–1.18	0.003
	Labour Agentry	0.90	0.84–0.95	<0.001
	Peritraumatic Dissociative Experiences			0.001
	Score < 22	—	—	
	Score ≥ 22	7.33	2.12–34.55	0.004

* FMI: Freiburg Mindfulness Inventory; TCI: Cloninger’s Temperament and Character Inventory; POMS: Profile of Mood Scale; STAI: State and Trait Anxiety Inventory; WEMWBS: Warwick-Edinburgh Mental Well-being Scale; SCL: Symptom Checklist; MPSS: Multidimensional Scale. — means that the modality in the row is the reference in the comparison with the other modalities of Perceived Social Support; QoL: Quality of Life; TES: traumatic event scale. ** VG was calculated as the average monthly scores.

**Table 6:** The final model resulting from the multivariate b selection procedure. In this procedure only variables were included that were significant in the univariate regression analyses.

Variables	OR *	CI 95% **	*p*-Value
FMI_acceptance (VI)	0.79	0.66–0.93	0.003
SCL_obsessive-compulsive (VI)	4.21	1.75–11.93	0.001
Having an history of psychological care (VI)	4.06	1.50–11.62	0.006

* OR: Odds ratios; ** CI: Confidence Interval, VI: visit at inclusion.

## Data Availability

The data presented in this study are available on request from the corresponding author. Data are not publicly available as they concern a specific population.

## References

[1] Martínez-Borba V., Suso-Ribera C., Osma J., Andreu-Pejó L. (2020). Predicting Postpartum Depressive Symptoms from Pregnancy Biopsychosocial Factors: A Longitudinal Investigation Using Structural Equation Modeling. Int. J. Environ. Res. Public Health.

[2] Woody C.A., Ferrari A.J., Siskind D.J., Whiteford H.A., Harris M.G. (2017). A systematic review and meta-regression of the prevalence and incidence of perinatal depression. J. Affect. Disord..

[3] World Health Organization (2008). Maternal Mental Health and Child Development in Low and Middle-Income Countries. www.who.int/mental_health/prevention/suicide/mmh_jan08_meeting_report.pdf.

[4] Slomian J., Honvo G., Emonts P., Reginster J.Y., Bruyère O. (2019). Consequences of maternal postpartum depression: A systematic review of maternal and infant outcomes. Women’s Health.

[5] Moraes G.P., Lorenzo L., Pontes G.A., Montenegro M.C., Cantilino A. (2017). Screening and diagnosing postpartum depression: When and how?. Trends Psychiatry Psychother..

[6] Loomans E.M., van Dijk A.E., Vrijkotte T.G., van Eijsden M., Stronks K., Gemke R.J., Van den Bergh B.R. (2013). Psychosocial stress during pregnancy is related to adverse birth outcomes: Results from a large multi-ethnic community-based birth cohort. Eur. J. Public Health.

[7] Meltzer-Brody S., Howard L.M., Bergink V., Vigod S., Jones I., Munk-Olsen T., Honikman S., Milgrom J. (2018). Postpartum psychiatric disorders. Nat. Rev. Dis. Primers.

[8] Simons S.S.H., Zijlmans M.A.C., Cillessen A.H.N., De Weerth C. (2019). Maternal prenatal and early postnatal distress and child stress responses at age 6. Stress.

[9] World Health Organization. https://www.who.int/mental_health/prevention/suicide/lit_review_postpartum_depression.pdf.

[10] Walsh K., Mccormack C.A., Webster R., Pinto A., Lee S., Feng T., Krakovsky H.S., O’Grady S.M., Tycko B., Champagne F.A. (2019). Maternal prenatal stress phenotypes associate with fetal neurodevelopment and birth outcomes. Proc. Natl. Acad. Sci. USA.

[11] Stewart D.E., Vigod S.N. (2019). Postpartum Depression: Pathophysiology, Treatment, and Emerging Therapeutics. Annu. Rev. Med..

[12] Eldar E., Rutledge R.B., Dolan R.J., Niv Y. (2016). Mood as Representation of Momentum. Trends Cogn. Sci..

[13] Raoult C.M.C., Moser J., Gygax L. (2017). Mood as Cumulative Expectation Mismatch: A Test of Theory Based on Data from Non-verbal Cognitive Bias Tests. Front. Psychol..

[14] Wong M.Y. (2016). Towards a theory of mood function. Philos. Psychol..

[15] Kreher J.B., Schwartz J.B. (2012). Overtraining syndrome: A practical guide. Sports Health.

[16] Vrijkotte S., Roelands B., Pattyn N., Meeusen R. (2019). The Overtraining Syndrome in Soldiers: Insights from the Sports Domain. Mil. Med..

[17] Sorenson D.L. (1990). Uncertainty in pregnancy. Naacog’s Clin. Issues Perinat. Women’s Health Nurs..

[18] Sevil Degirmenci S., Kosger F., Altinoz A.E., Essizoglu A., Aksaray G. (2020). The relationship between separation anxiety and intolerance of uncertainty in pregnant women. J. Matern.-Fetal Neonatal Med..

[19] Sperry S.H., Walsh M.A., Kwapil T.R. (2020). Emotion dynamics concurrently and prospectively predict mood psychopathology. J. Affect. Disord..

[20] Bei B., Coo S., Trinder J. (2015). Sleep and Mood during Pregnancy and the Postpartum Period. Sleep Med. Clin..

[21] Hillerer K.M., Neumann I.D., Slattery D.A. (2012). From stress to postpartum mood and anxiety disorders: How chronic peripartum stress can impair maternal adaptations. Neuroendocrinology.

[22] Kabat-Zinn J. (1994). Wherever You Go, There You Are.

[23] Grossman P., Niemann LSchmidt S., Walach H. (2004). Mindfulness-based stress reduction and health benefits. A meta-analysis. J. Psychosom. Res..

[24] Lutz A., Jha APDunne J.D., Saron C.D. (2015). Investigating the Phenomenological Matrix of Mindfulness-related Practices from a Neurocognitive Perspective. Am. Psychol..

[25] Walach H., Buchheld N., Buttenmüller V., Kleinknecht N., Schmidt S. (2006). Measuring mindfulness—the Freiburg mindfulness inventory (FMI). Pers. Individ. Differ..

[26] Hayes A.M., Feldman G. (2004). Clarifying the construct of mindfulness in the context of emotion regulation and the process of change in therapy. Clin. Psychol. Sci..

[27] Baer R.A., Smith G.T., Hopkins J., Krietemeyer L., Toney L. (2006). Using self-report assessment methods to explore facets of mindfulness. Assessment.

[28] Brown K.W., Ryan R.M. (2003). The benefits of being present: Mindfulness and its role in psychological well-being. J. Pers. Soc. Psychol..

[29] Hanley A.W., Garland E.L. (2017). The mindful personality: A meta-analysis from a cybernetic perspective. Mindfulness.

[30] Barnhofer T., Duggan D.S., Griffith J.W. (2011). Dispositional mindfulness moderates the relation between neuroticism and depressive symptoms. Pers. Individ. Differ..

[31] Giluk T.L. (2009). Mindfulness, Big Five personality, and affect: A meta-analysis. Pers. Individ. Differ..

[32] Hulsbosch L.P., Boekhorst M.G.B.M., Potharst E.S., Pop V.J.M., Nyklíček I. (2021). Trait mindfulness during pregnancy and perception of childbirth. Arch. Women’s Ment. Health.

[33] Baer R.A. (2006). Mindfulness training as a clinical intervention: A conceptual and empirical review. Clin. Psychol. Sci. Pract..

[34] Carmody J., Baer R.A. (2008). Relationships between mindfulness practice and levels of mindfulness, medical and psychological symptoms and well-being in a mindfulness-based stress reduction program. J. Behav. Med..

[35] Hofmann S.G., Sawyer A.T., Witt A.A., Oh D. (2010). The effect of mindfulness-based therapy on anxiety and depression: A meta-analytic review. J. Consult. Clin. Psychol..

[36] Freudenthaler L., Turba J.D., Tran U.S. (2017). Emotion regulation mediates the associations of mindfulness on symptoms of depression and anxiety in the general population. Mindfulness.

[37] Hall H.G., Beattie J., Lau R., East C., Anne Biro M. (2016). Mindfulness and perinatal mental health: A systematic review. Women Birth.

[38] Matvienko-Sikar K., Lee L., Murphy G., Murphy L. (2016). The effects of mindfulness interventions on prenatal well-being: A systematic review. Psychol. Health.

[39] Sun Y., Li Y., Wang J., Chen Q., Bazzano A.N., Cao F. (2021). Effectiveness of Smartphone-Based Mindfulness Training on Maternal Perinatal Depression: Randomized Controlled Trial. J. Med. Internet Res..

[40] Woolhouse H., Mercuri K., Judd F., Brown S.J. (2014). Antenatal mindfulness intervention to reduce depression, anxiety and stress: A pilot randomised controlled trial of the MindBabyBody program in an Australian tertiary maternity hospital. BMC Pregnancy Childbirth.

[41] Cox J.L., Holden J.M., Sagovsky R. (1987). Detection of postnatal depression. Development of the 10-item Edinburgh Postnatal Depression Scale. Br. J. Psychiatry.

[42] Guedeney N., Fermanian J. (1998). Validation study of the French Version of the Edinburgh Postnatal Depression Scale (EPDS): New results about use and psychometric properties. Eur. Psychiatry.

[43] Trousselard M., Steiler D., Raphel C., Cian C., Duymedjian R., Claverie D., Canini F. (2010). Validation of a French version of the Freiburg Mindfulness Inventory-short version: How mindfulness deals with the stress in a working middle-aged opulation. Biopsychosoc. Med..

[44] Shacham S. (1983). A shortened version of profile of mood states. J. Pers. Assess..

[45] Fillion L., Gagnon P. (1999). French Adaptation of the Shortened Version of the Profile of Mood States. Psychol. Rep..

[46] Cloninger C.R., Svrakic D.M., Przybeck T.R. (1993). A psychobiological model of temperament and character. Arch. Gen. Psychiatry.

[47] Cloninger C.R., Svrakic N.M., Svrakic D.M. (1997). The Role of personality self-organization in development of mental order and disorder. Dev. Psychopathol..

[48] Adan A., Serra-Grabulosa J.M., Natale V. (2009). A reduced Temperament and Character Inventory (TCI-56). Psychometric properties in a non-clinical sample. Pers. Individ. Differ..

[49] Spielberger C. (1983). Manual for the State-Trait-Anxiety Inventory: STAI (Form Y).

[50] Spielberger C.D., Smith L.H. (1966). Anxiety (drive), stress, and serial-position effects in serial-verbal learning. J. Exp. Psychol..

[51] Tennant R., Hiller L., Fishwick R., Platt S., Joseph S., Weich S., Parkinson J., Secker J., Stewart-Brown S. (2007). The Warwick-Edinburgh Mental Well-being Scale (WEMWBS): Development and UK validation. Health Qual. Life Outcomes.

[52] Trousselard M., Canini F., Dutheil F., Claverie D., Fenouillet F., Naughton G., Steward-Brown S., Franck N. (2016). Investigating well-being in healthy population and schizophrenia with the WEMWBS. Psychiatry Res..

[53] Derogatis L.R., Lipman R.S., Rickels K., Uhlenhuth E.H., Covi L. (1974). The Hopkins Symptom Checklist (HSCL): A self-report symptom inventory. Behav. Sci..

[54] Muller M.E. (1993). Development of the Prenatal Attachment Inventory. West J. Nurs. Res..

[55] Foley S., Hughes C. (2018). Great expectations? Do mothers’ and fathers’ prenatal thoughts and feelings about the infant predict parent-infant interaction quality? A meta-analytic review. Dev. Rev..

[56] Righetti-Veltema M., Conne-Perréard E., Bousquet A., Manzano J. (2007). Construction et validation multicentrique d’un questionnaire prépartum de dépistage de la dépression postpartum. Psychiatr. Enfant..

[57] Zimet G.D., Dahlem N.W., Zimet S.G., Farley G.K. (1988). The Multidimensional Scale of Perceived Social Support. J. Pers. Assess..

[58] Denis A., Callahan S., Bouvard M. (2015). Evaluation of the French version of the Multidimensional Scale of Perceived Social Support during the postpartum period. Matern. Child Health J..

[59] Hodnett E.D., Simmons-Tropea D.A. (1987). The Labour Agentry Scale: Psychometric properties of an instrument measuring control during childbirth. Res. Nurs. Health.

[60] Wijma K., Söderquist J., Wijma B. (1997). Posttraumatic stress disorder after childbirth: A cross sectional study. J. Anxiety Disord..

[61] American Psychiatric Association (1994). Diagnostic and Statistical Manual of Mental Disorders.

[62] Marmar C.R., Weiss D.S., Metzler T.J., Wilson J.P., Marmar C.R. (1997). The Peritraumatic Dissociative Experiences Questionnaire. Assessing Psychological Trauma and Posttraumatic Stress Disorder.

[63] Birmes P., Brunet A., Benoit M., Defer S., Hatton L., Sztulman H., Schmitt L. (2005). Validation of the Peritraumatic Dissociative Experiences Questionnaire self-report version in two samples of French-speaking individuals exposed to trauma. Eur. Psychiatry.

[64] Birmes P., Carreras D., Ducassé J.-L., Charlet J.-P., Warner B.A., Lauque D., Schmitt L. (2001). Peritraumatic Dissociation, Acute Stress, and Early Posttraumatic Stress Disorder in Victims of General Crime. Can. J. Psychiatry.

[65] Kohls N., Sauer S., Walach H. (2009). Facets of mindfulness–Results of an online study investigating the Freiburg mindfulness inventory. Pers. Individ. Differ..

[66] Menges J., Caltabiano M. (2019). The effect of mindfulness on academinc self-efficcacy: A randomosed controlled trial. Int. J. Educ. Psychol. Couns..

[67] Hayes S.C., Luoma J.B., Bond F.W., Masuda A., Lillis J. (2006). Acceptance and commitment therapy: Model, processes and outcomes. Behav. Res. Ther..

[68] Neff K. (2003). Self-Compassion: An Alternative Conceptualization of a Healthy Attitude Toward Oneself. Self Identity.

[69] Arch J.J., Brown K.W., Dean D.J., Landy L.N., Brown K., Laudenslager M.L. (2014). Self-compassion training modulates alpha-amylase, heart rate variability, and subjective responses to social evaluative threat in women. Psychoneuroendocrinology.

[70] Brown L., Bryant C., Brown V., Bei B., Judd F. (2016). Self-compassion, attitudes to ageing and indicators of health and well-being among midlife women. Aging Ment. Health.

[71] Ford J., Klibert J.J., Tarantino N., Lamis D.A. (2017). Savouring and Self-compassion as Protective Factors for Depression: Protective Factors for Depression. Stress Health.

[72] Sauer S., Walach H., Schmidt S., Hinterberger T., Horan M., Kohls N. (2011). Implicit and explicit emotional behavior and mindfulness. Conscious. Cogn..

[73] Russell E.J., Fawcett J.M., Mazmanian D. (2013). Risk of obsessive compulsive disorder in pregnant and postpartum women: A meta-analysis. J. Clin. Psychiatry.

[74] Sharma V., Sommerdyk C. (2015). Obsessive–compulsive disorder in the postpartum period: Diagnosis, differential diagnosis and management. Women’s Health.

[75] Findley D.B., Leckman J.F., Katsovich L., Lin H., Zhang H., Grantz H., Otka J., Lombroso P.J., King R.A. (2003). Development of the Yale Children’s Global Stress Index (YCGSI) and its application in children and adolescents with Tourette’s syndrome and obsessive-compulsive disorder. J. Am. Acad. Child Adolesc. Psychiatry.

[76] Toro J., Cervera M., Osejo E., Salamero M. (1992). Obsessive-compulsive disorder in childhood and adolescence: A clinical study. J. Child Psychol. Psychiatry.

[77] Goodwin G.M. (2015). The overlap between anxiety, depression, and obsessive-compulsive disorder. Dialogues Clin. Neurosci..

[78] Miller E.S., Chu C., Gollan J., Gossett D.R. (2013). Obsessive-compulsive symptoms during the postpartum period. A prospective cohort. J. Reprod. Med..

[79] Van Broekhoven K.E.M., Karreman A., Hartman E.E., Lodder P., Endendijk J.J., Bergink V., Pop V.J.M. (2019). Obsessive-compulsive personality disorder symptoms as a risk factor for postpartum depressive symptoms. Arch. Women’s Ment. Health.

[80] Barcaccia B., Tenore K., Mancini F. (2015). Early childhood experiences shaping vulnerability to Obsessive-Compulsive Disorder. Clin. Neuropsychiatry.

[81] Yorulmaz O., Gençöz T., Woody S. (2010). Vulnerability factors in OCD symptoms: Cross-cultural comparisons between Turkish and Canadian samples. Clin. Psychol. Psychother..

[82] Lord C., Hall G., Soares C.N., Steiner M. (2011). Physiological stress response in postpartum women with obsessive—Compulsive disorder: A pilot study. Psychoneuroendocrinology.

[83] Musser E.D., Ablow J.C., Measelle J.R. (2012). Predicting maternal sensitivity: The roles of postnatal depressive symptoms and parasympathetic dysregulation. Infant Ment. Health J..

[84] Goleman D. (1996). Emotional Intelligence: Why It Can Matter More than IQ.

[85] Balsamo M. (2010). Anger and depression: Evidence of a possible mediating role for rumination. Psychol. Rep..

[86] Ascenzo N., Collard J.J. (2018). Anger, forgiveness, and depression in the postnatal experience. Ment. Health Fam. Med..

[87] Bruno A., Laganà A.S., Leonardi V., Greco D., Merlino M., Vitale S.G., Triolo O., Zoccali R.A., Muscatello M.R.A. (2018). Inside–out: The role of anger experience and expression in the development of postpartum mood disorders. J. Matern. Fetal. Neonatal. Med..

[88] Ou C.H., Hall W.A. (2018). Anger in the context of postnatal depression: An integrative review. Birth.

[89] Tobe H., Kita S., Hayashi M., Umeshita K., Kamibeppu K. (2020). Mediating effect of resilience during pregnancy on the association between maternal trait anger and postnatal depression. Compr. Psychiatry.

[90] Bishop S.R., Lau M., Shapiro S., Carlson L., Anderson N.D., Carmody J., Segal Z.V., Abbey S., Speca M., Velting D. (2004). Mindfulness: A proposed operational definition. Clin. Psychol..

[91] Gillespie S.M., Garofalo C., Velotti P. (2018). Emotion regulation, mindfulness, and alexithymia: Specific or general impairments in sexual, violent, and homicide offenders?. J. Crim. Justice.

[92] Garofalo C., Gillespie S.M., Velotti P. (2020). Emotion regulation mediates relationships between mindfulness facets and aggression dimensions. Aggress. Behav..

[93] Cludius B., Mannsfeld A.K., Schmidt A.F., Jelinek L. (2021). Anger and aggressiveness in obsessive–compulsive disorder (OCD) and the mediating role of responsibility, non-acceptance of emotions, and social desirability. Eur. Arch. Psychiatry Clin. Neurosci..

[94] Kabat-Zinn J., Lipworth L., Burney R. (1985). The Clinical Use of Mindfulness Meditation for the Self-Regulation of Chronic Pain. J. Behav. Med..

[95] Chiesa A., Serretti A. (2009). Mindfulness-based stress reduction for stress management in healthy people: A review and meta-analysis. J. Altern. Complement. Med..

[96] Lönnberg G., Nissen E., Niemi M. (2018). What is learned from Mindfulness Based Childbirth and Parenting Education?—Participants’ experiences. BMC Pregnancy Childbirth.

[97] Kabat-Zinn J. (1982). An outpatient program in behavioral medicine for chronic pain patients based on the practice of mindfulness meditation: Theoretical considerations preliminary results. Gen. Hosp. Psychiatry.

[98] Segal Z.V., Williams J.M.G., Teasdale J.D. (2022). Mindfulness-Based Cognitive Therapy for Depression: A New Approach to Preventing Relapse.

[99] Flaxman P.E., Bond F.W., Baer R.A. (2006). Acceptance and Commitment Therapy (ACT) in the Workplace. Mindfulness-Based Treatment Approaches: Clinician’s Guide to Evidence Base and Applications.

